# Response dynamics of rat barrel cortex neurons to repeated sensory stimulation

**DOI:** 10.1038/s41598-017-11477-6

**Published:** 2017-09-13

**Authors:** Ehsan Kheradpezhouh, Mehdi Adibi, Ehsan Arabzadeh

**Affiliations:** 1 Eccles Institute of Neuroscience, John Curtin School of Medical Research, Australian National University, Canberra, ACT Australia; 2 Australian Research Council Centre of Excellence for Integrative Brain Function, Australian National University Node, Canberra, ACT Australia; 3 University of New South Wales, UNSW, Sydney, NSW Australia; 4 International School for Advanced Studies – SISSA, Trieste, Italy

## Abstract

Neuronal adaptation is a common feature observed at various stages of sensory processing. Here, we quantified the time course of adaptation in rat somatosensory cortex. Under urethane anesthesia, we juxta-cellularly recorded single neurons (n = 147) while applying a series of whisker deflections at various frequencies (2–32 Hz). For ~90% of neurons, the response per unit of time decreased with frequency. The degree of adaptation increased along the train of deflections and was strongest at the highest frequency. However, a subset of neurons showed facilitation producing higher responses to subsequent deflections. The response latency to consecutive deflections increased both for neurons that exhibited adaptation and for those that exhibited response facilitation. Histological reconstruction of neurons (n = 45) did not reveal a systematic relationship between adaptation profiles and cell types. In addition to the periodic stimuli, we applied a temporally irregular train of deflections with a mean frequency of 8 Hz. For 70% of neurons, the response to the irregular stimulus was greater than that of the 8 Hz regular. This increased response to irregular stimulation was positively correlated with the degree of adaptation. Altogether, our findings demonstrate high levels of diversity among cortical neurons, with a proportion of neurons showing facilitation at specific temporal intervals.

## Introduction

Exposure of sensory neurons to repeated stimulation results in changes in neuronal response properties over time – a phenomenon known as sensory adaptation. Adaptation is sometimes characterized in terms of an attenuation of neuronal responsiveness to the repeated sensory stimulation. However, the current view of adaptation is a continuous recalibration of the sensory system to compensate for the changes in the statistics of the input stream^1–9^. According to this view, adaptation affects information processing by producing shifts in the neuronal input-output relation^10,11^. In the whisker sensory pathway, neuronal response adaptation has been quantified along the various stages of processing, from first order neurons in the trigeminal ganglion to brainstem, sensory thalamic nuclei and across the layers of the somatosensory cortex^12–20^. In the vibrissal primary somatosensory cortex (vS1), application of repetitive whisker stimulation is found to reduce neuronal responses both at the level synaptic input and spiking activity^12,14,17–19,21–23^.

The degree of adaptation depends on stimulus parameters including frequency, duration and amplitude^12,19,24–26^. Typically, as the stimulation frequency increases, neurons adapt at a faster rate^17,27^. However, the effect of stimulus amplitude on adaptation is complex and the degree of adaptation does not monotonically increase with stimulus amplitude^14^. Beyond the parameters of stimulation, the neuron’s location within the cortical circuit and its intrinsic properties such as its cell type are expected to influence its adaptation profile^12–14,17,19,28,29^. There is however a high level of diversity in adaptation among neurons throughout the vibrissal sensory pathway from brain stem to thalamus and cortex^30,31^. In addition to its effects at the level of individual neurons, adaptation has been shown to influence the responses at the network level by (i) modulating the correlations amongst neurons^9, 24,32,33^, and (ii) reducing the network heterogeneity in rat vS1 cortex^34^. Similar homeostatic effects have been observed in the primary visual cortex of anesthetized cats^35^ where adaptation decorrelated neurons and maintained their population responding rate. This evidence suggests that along with the intrinsic properties of individual neurons^36–38^, the network properties play a key role in the dynamics of sensory adaptation.

Here, we applied a series of brief whisker deflections of constant amplitude to produce cortical responses to discrete stimuli with well-defined time course and quantified the profile of adaptation for individual neurons recorded across layers of the vS1 cortex. The focus on temporal patterns allows us to examine how individual neurons adapt to different temporal aspects of a discrete sequence of deflections, such as its rate and regularity. To quantify the temporal profile of adaptation, we applied the deflections at various frequencies ranging from 2 Hz to 32 Hz. This frequency range includes the range of frequencies at which rodents sweep their whiskers back and forth to gather tactile information from their surrounding environment^21,39,40^ as well as the higher frequencies of foveal whisking^41^. The discrete nature of deflections also simulates the abrupt and transient stick-slip events, which occur when whiskers make contact with textured surfaces^42^. As the frequency of stimulation increases, the net neuronal response is expected to reflect a tradeoff: on the one hand increasing the number of stimulations is predicted to enhance the overall evoked response and on the other hand, the stronger adaptation at higher frequencies is predicted to reduce the response over time. Here, we quantify how this tradeoff determines the frequency at which the neurons elicit their maximum response.

## Results

**Figure 1:**
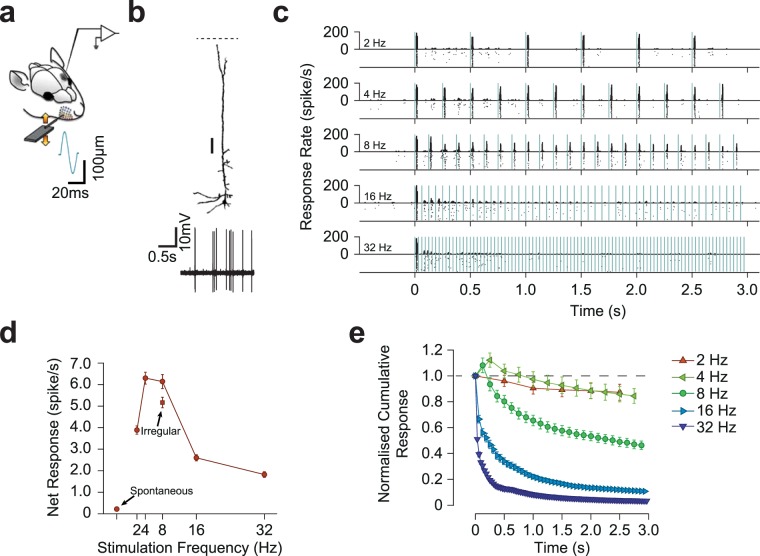
Stimulation paradigm and characterization of the adaptation profile. (**a**) Single neurons were juxtacellularly recorded from vS1 cortex of anesthetized rats while applying deflections to the principal whisker. (**b**) Histological reconstruction of a sample layer 5 pyramidal neuron. Scale bar, 100 µm. (**c**) Raster plots and PSTHs for the same neuron as in (**b**) for different stimulation frequencies. Vertical green lines indicate individual deflections (200 µm in amplitude). Dots in the lower parts of panels indicate individual spikes and rows correspond to trials. (**d**) The net response rate of the neuron in B during the 3-s stimulation period. (**e**) The cumulative response of the sample neuron as a function of time normalized to the response to the first deflection. Error bars indicate standard error of the means across trials.

To quantify the response dynamics of cortical neurons to repeated sensory stimulation, we employed loose cell-attached recording from individual neurons across layers of the vS1 cortex. We applied a 3-s train of discrete deflections of 200-μm amplitude to the neuron’s principal whisker at frequencies of 2, 4, 8, 16 and 32 Hz. Figure 1 illustrates the response profile of a layer 5 pyramidal neuron and summarizes how we quantify the degree of adaptation to the stimulus train. The loose cell-attached recording method provides reliable identification of individual spikes due to its high signal to noise ratio (Fig. 1a) and further allows labeling the neurons (n = 45) and identifying their morphology.

### Profound adaptation of cortical neurons to repeated whisker stimulation

The adaptation of the response profile of the sample pyramidal neuron is evident in the raster plot and peri-stimulus time histograms (PSTHs) shown in Fig. 1c. At lower stimulation frequencies (2 and 4 Hz), the response to consecutive deflections remained relatively unchanged revealing minimal adaptation. As the rate of stimulation increased to 8 Hz and higher frequencies, the response to later deflections diminished revealing prominent adaptation. To better quantify the effect of adaptation, we measured the net neuronal response rate within the whole stimulus duration (3 s) averaged across trials. For this sample neuron, the net response was maximum for the stimulation frequency of 4 Hz at which the effect of adaptation was minimal (Fig. 1d). At frequencies above 8 Hz, profound adaptation reduced the neuronal response over time. This decrease in the net neuronal response was observed despite the fact that at higher frequencies the neuron’s principal whisker was stimulated with a higher number of deflections during the 3-s trial duration (96 deflections at 32 Hz compared to 6 deflections at 2 Hz). We further quantified the changes in the responsiveness as a function of time for every stimulation frequency in terms of the cumulative response rate relative to the first deflection. The sample pyramidal neuron exhibits a systematic decrease in responsiveness with time (Fig. 1e). The decline was steeper and reached a lower level as the stimulation frequency increased indicating a faster and increased adaptation with stimulation frequency.

**Figure 2:**
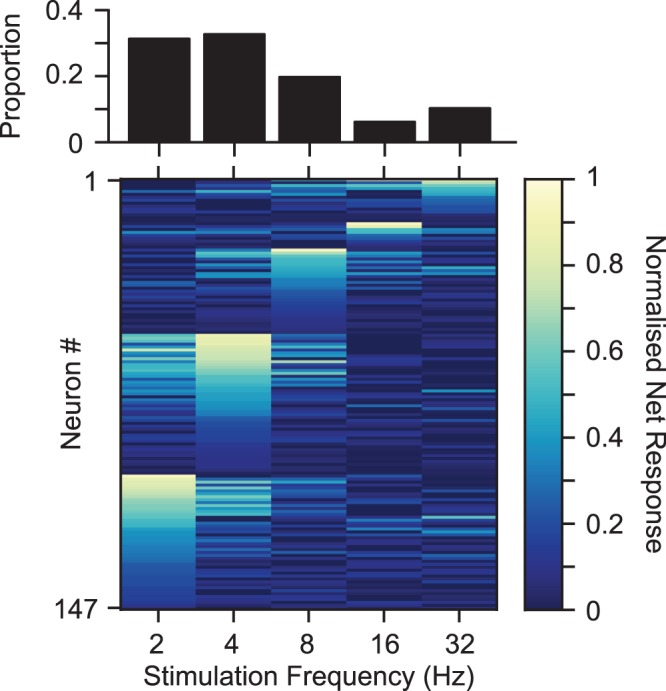
The net neuronal response to different frequencies. Each row corresponds to the net response of an individual neuron over the 3 s duration of stimulation normalized to the maximum response across frequencies. Neurons were sorted based on the frequency to which they produced the maximum net response (2 Hz at the bottom and 32 Hz at the top). The upper panel illustrates the histogram of frequencies at which the net response was maximum.

Figure 2 shows how the observed decline in the net neuronal response generalizes to all recorded neurons (n = 147). For over 90% of neurons, the maximum net response was observed at frequencies other than the maximum stimulation frequency (i.e. 32 Hz). The observation that the maximum deflection frequency did not elicit the maximum neuronal response is consistent with previous recordings in vS1 cortex^12,26,43–46^.

**Figure 3:**
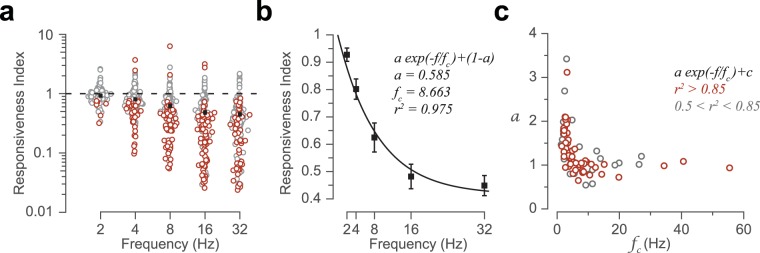
Adaptation increased with stimulation frequency. (**a**) Every circle corresponds to an individual neuron. Red circles indicate neurons with statistically significant (permutation test, *p* < 0.05) adaptation (responsiveness index < 1) or facilitation (responsiveness index > 1). Black squares represent the mean responsiveness index across neurons. (**b**) The profile of adaptation as a function of frequency. The square markers are as in (**a**). Error bars represent the standard error of the means across neurons. The curve corresponds to the exponential function that best fits the data. (**c**) The parameters of the best fit of an exponential function for individual neurons; change in responsiveness is denoted by a, and the frequency constant of the decay is denoted by f_c_. Each marker indicates a single neuron. Colors correspond to different levels of goodness of the fit.

In order to quantify the degree of adaptation for individual neurons at each frequency, we defined the following indices. The *responsiveness index* was defined as the net neuronal response rate to the 3-s train of deflections divided by the rate of spiking in response to the first deflection. Likewise, the *cumulative responsiveness index* was defined as the cumulative response rate as a function of time normalized to the rate of response to the first deflection. A responsiveness index of less than one indicates the proportion of response attenuation relative to the initial response to the first deflection (i.e. response adaptation). A value greater than one, however indicates response facilitation over the time course of stimulation. When the neuronal response to consecutive deflections does not exhibit any systematic variation over time (e.g. little adaptation or facilitation), responsiveness index is close to one. Figure 3 shows the responsiveness index for all recorded neurons. On average, across all neurons, at 2 Hz stimulation the index was 0.93 revealing minimal adaptation. By increasing the stimulation frequency, the average responsiveness index gradually declined to 0.43 at 32 Hz (Fig. 3). This declining profile of the responsiveness index was well fit by an exponential function (Fig. 3b). To quantity the diversity of the adaptation profile across neurons, we further fit a similar function as in Fig. 3b on data from individual neurons (Fig. 3c). The fit well characterized the adaptation profile for 76 neurons (51.7%, *r*^2^ > 0.5). For 53 of these neurons with *r*^2^ > 0.85, the corresponding frequency constant of the exponential drop was 4.4 Hz (interquartile range: 2.8 to 8.4 Hz).

**Figure 4:**
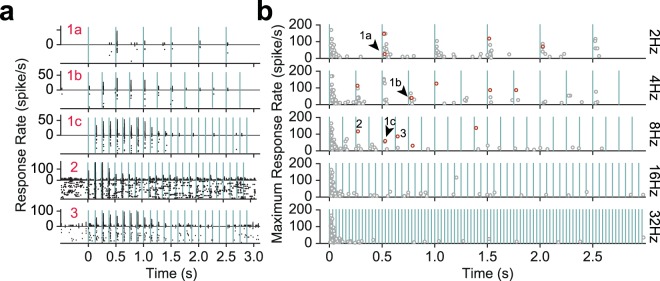
Prominent response facilitation in a subset of neurons. (**a**) Response profile of three sample neurons exhibiting response facilitation. The conventions are the same as in Fig. 1c. (**b**) Maximum response over the course of stimulation as a function of the time instance of the maximum response. Each data point corresponds to a neuron. Red circles indicate neurons for which the maximum response to a later subsequent deflection was significantly greater than the response to the first deflection (based on permutation test, *p* < 0.05). Arrows indicate the three sample neurons from (**a**).

For a subset of recorded neurons, the responsiveness index was greater than 1, indicative of response facilitation. Figure 4a depicts the raster plots and PSTHs for three sample neurons exhibiting prominent response facilitation. At 2 Hz stimulation, neuron 1 exhibits no evoked activity in response to the first deflection, but produces a reliable evoked activity 500 ms later in response to the second deflection. The response profile at 4 and 8 Hz further reveal a systematic facilitation in response of this neuron. Similarly, neurons 2 and 3 exhibit their highest evoked response not to the first deflection, but to subsequent deflections in the stimulation train. Figure 4b identifies the instances of neuronal facilitation, by illustrating for each neuron the deflection that produced the maximum neuronal response. The magnitude of the maximum response is captured in the y-axis. A proportion of neurons exhibited their maximum response rate not to the first deflection but to later subsequent deflections, indicative of response facilitation (red circles indicate neurons for which facilitation was statistically significant based on a permutation test, *p* < 0.05).

### Response latencies increase during the stimulation train

**Figure 5:**
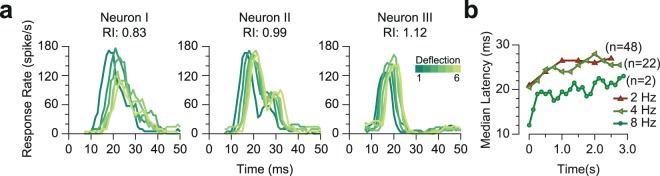
Response latencies increased over the time course of stimulation. (**a**) PSTHs for 3 sample neurons for 6 deflections at 2 Hz stimulation. Different shades of green represent the order of deflection within the simulation train, with darker corresponding to earlier deflections. The three neurons are selected as examples of an adapted neuron (left panel, responsiveness index of 0.83), a facilitated neuron (right panel, responsiveness index of 1.12) and a neuron with little adaptation or facilitation at 2 Hz (middle panel, responsiveness index of 0.99). (**b**) Median response latencies to individual deflections for neurons that exhibited a significant evoked response to all deflections.

Apart from the prominent response adaptation, the sample pyramidal neuron in Fig. 1 exhibits a systematic increase in the latency of responses to subsequent deflections. Figure 5 quantifies the response latencies at early versus late deflections in the stimulation train. Across neurons, response latency increased over the time course of stimulation, irrespective of the dynamics of their response rate (facilitation versus adaptation).

### Temporal regularity and adaptation

**Figure 6:**
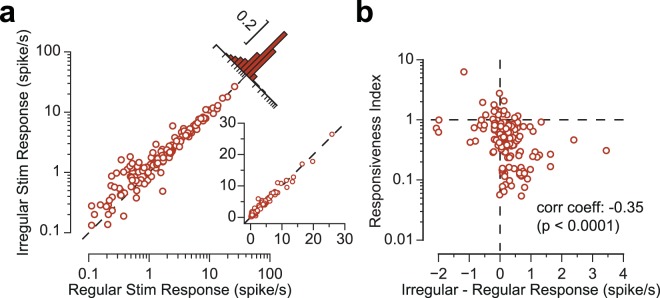
Response to regular versus irregular train of deflections. (**a**) The net neuronal response to the irregular train (mean frequency of 8 Hz) is plotted against the net neuronal response to the regular stimulation at 8 Hz. Every circle represents a single neuron. The inset represents the same data but in linearly scaled axes. (**b**) As in (**a**), every dot represents one neuron. Overall, 70% of neurons produced higher response to the irregular stimulus (are located to the right of the vertical dashed line). The heightened response to irregular stimulation was significantly correlated with the responsiveness index.

To what extent does the precise timing of the deflections within a train determine the degree of sensory adaptation? To address this question, in addition to the regular stimulations, we applied a temporally irregular train of deflections with a mean frequency of 8 Hz. For 70% of neurons, the response to the irregular stimulus was greater than that to the regular stimulation (Fig. 6a). This finding is consistent with previous research indicating higher activity in response to temporally noisy stimulation trains^47,48^. To quantify the extent to which the degree of adaptation depends on the stimulus regularity, Fig. 6b illustrates the relationship between responsiveness index and the difference in activity between regular and irregular stimulation. We observed a negative correlation between responsiveness index and the response difference between irregular stimulation and regular stimulation (−0.3451, *p* < 0.0001). This correlation indicates that neurons that adapt more strongly to 8 Hz, exhibit a greater difference in their net response to irregular stimulation versus the regular one. Thus, the physiological mechanism that leads to strong adaptation seems to be sensitive to the irregularity of input, such that the adapting force is boosted by a regular stimulus.

### The diversity of response dynamics across neurons

**Figure 7:**
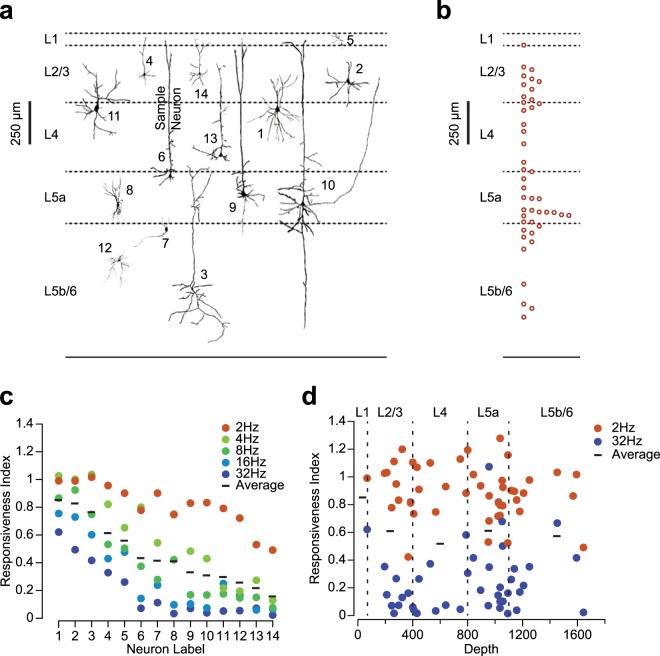
Neuronal reconstruction and diversity of adaptation. (**a**) The morphology of 14 example reconstructed neurons illustrated in their cortical location as identified by histology. The sample neuron from Fig. 1 is marked as number 6. (**b**) Soma positions for the 45 reconstructed neurons. (**c**) The responsiveness index for the 14 neurons from panel (**a**). The horizontal dashed line indicates the index for each neuron averaged across all frequencies. (**d**) The responsiveness index as a function of depth for all reconstructed neurons.

Despite the systematic drop in the responsiveness index with stimulation frequency, we observed a high level of diversity among neurons in their degree and profile of adaptation (Fig. 3a and c). We therefore investigated what functional and morphological properties of the recorded neurons account for this high level of diversity. Juxtacellular labelling^49^ and histological reconstruction allowed us to obtain the morphology and topographical information of a subset of neurons. Neuronal reconstruction did not reveal any evident relationships between cell morphology and the neurons’ response profile to repeated stimulation. For instance, two pairs of neurons with similar morphology (neurons 1 and 11, and neurons 4 and 14 in Fig. 7a) showed different adaptation profiles (see Fig. 7c). On the other hand, neurons with similar responsiveness index could have distinct morphologies (neurons 8, 9 and 10 in Fig. 7a). We next examined the responsiveness index across layers. As neurons could potentially be recorded by a contact between the pipette tip and any segment of the neuron, the depth of the recording does not necessarily represent the location of the soma. We therefore limited our analysis to neurons that had a morphological reconstruction (Fig. 7b). For these neurons, Fig. 7d captures the adaption index for the lowest stimulus frequency (2 Hz), the highest stimulus frequency (32 Hz) and the mean adaption index across all frequencies, as a function of depth. Again, we found high level of diversity and no evident correlation between responsiveness index and the depth at which the neuron was recorded.

**Figure 8:**
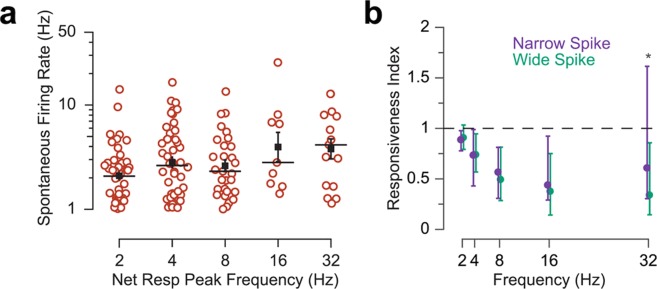
Correlation between the neurons’ baseline firing rate and adaptation. (**a**) Every circle represents a single neuron. For each neuron, the y-axis plots the spontaneous firing rate, and the x-axis indicates the frequency that produced the highest net evoked response. The net response is defined as firing rate during the 3 s stimulation period. Neurons with higher spontaneous rates tended to have their peak evoked response at higher frequencies. (**b**) The responsiveness index as a function of frequency for two classes of narrow-spike (purple) and wide-spike neurons (cyan). The circles correspond to the median responsiveness of each category, and error bars indicate the interquartiles, *p < 0.05.

Finally, we examined the link between adaptation and the physiological properties of the neurons. We first characterized the relationship between adaptation and the spontaneous firing rate of neurons. A neuron that exhibits minimum degree of adaptation is expected to produce its maximum response at the highest stimulation frequency. For each neuron, we therefore identified the stimulus frequency that elicited the maximum net evoked response. Figure 8a reveals that neurons with higher spontaneous firing rate tended to have the peak of their net response at higher stimulation frequencies (i.e. exhibited lower degrees of adaptation).

In the barrel cortex, the width of a neuron’s action potential has been used as an indication of cell type^45^. We categorized our recorded neurons into two classes of narrow spike-waveform, putative inhibitory interneurons and wide spike-waveform, putative excitatory neurons. Neurons with a narrow waveform on average exhibited a higher level of responsiveness (lower adaptation) at 32 Hz frequency compared to those with a wide waveform (Wilcoxon rank-sum test; *p* < 0.05, Fig. 8b).

In conclusion, our findings demonstrate a high level of diversity among cortical neurons in their response to a train of stimulation, with a small proportion of neurons showing facilitation at specific temporal intervals.

## Discussion

The rodent vibrissal system provides a suitable model to study neuronal coding efficiency and adaptation due to its functional efficiency^50^ and structural organization^51–53^. Here, we examined how adaptation in vS1 cortex depends on the temporal properties of a series of discrete stimuli. We performed juxtacellular recordings from vS1 neurons and characterized the time course of adaptation. The majority of recorded neurons exhibited profound adaptation whereby the net neuronal response to the whole train of deflections decreased with stimulation frequency. This was a result of a gradual decrease in responsiveness to individual deflections, which exponentially decayed with time. However, in a small subset of neurons, we observed strong response facilitation over time, where sustained stimulation significantly increased the response to subsequent stimulations. Additionally, across all neurons, response latency increased over the time course of stimulation, and this was true both for neurons that exhibited profound adaptation and those that exhibited response facilitation.

As nocturnal animals, rodents explore their environment by whisking against surfaces and objects at frequencies ranging 5–25 Hz^41,54^. At the whisker follicle, the mechanoreceptors convert the movement into neuronal signals, which are transferred through trigeminal ganglion, brain stem and thalamus to the cortex for processing. Neuronal response adaptation has been observed at all stages of processing^12,14–19^. The degree of adaptation depends mainly on the frequency of whisker stimulation^12,17–19^, its intensity (in terms of its amplitude and velocity)^14,15^, and the cortical state^55,56^. Consistent with previous studies, we found that for most of the recorded neurons (~90%), the evoked response decreased from the first deflection to a steady-state response level^12,17,21,22,45,56^. However, for a small subset of neurons, the evoked response was facilitated during the stimulus train. This finding is consistent with previous studies showing facilitation of responses at stimulation frequencies < 10 Hz^30,57,58^. Facilitation of neuronal response is also reported in the visual system^11^ where facilitation can be linked to the center-surround structure of receptive fields. Repeated stimulation can reduce the surround suppression resulting in a disinhibition of the neuronal response to subsequent stimulations^59^. It is likely that the interaction between the excitatory and inhibitory inputs and their adaptation contributes to the facilitation observed in the subset vS1 neurons.

In the somatosensory system, the balance between excitatory and inhibitory inputs can shape the neuronal response profile and the degree of adaptation^18^. In the vS1 cortex, the reduction in neuronal response to the repetitive whisker stimulation is attributed to an alteration of the excitatory-inhibitory balance^18^. The imbalance between excitatory and inhibitory signals is also implicated in the post-adaptation facilitation observed in L4 pyramidal neurons of vS1^60^. However, repetitive whisker stimulations can produce significant response adaptation even in the absence of any detectable change in the balance between excitatory and inhibitory signals^22^. A key mechanism underlying adaptation is the short-term synaptic depression (STD) observed at the thalamocortical synapses^20,61,62^. Further experiments are required to investigate the synaptic and network mechanisms responsible for the response facilitation.

Besides stimulus properties, the intrinsic properties of a neuron including its location in the cortex and cell type can contribute to the neuron’s adaptation profile^21,30,63,64^. As the stimulus signals are transferred from whisker follicles to the cortex, the degree of adaptation systematically increases at consecutive stages of processing^14,17,19,21,29^. Within the vS1 cortex, there is a high level of diversity in adaptation^63,64^. This diversity of adaptation could be due to differences in the excitatory and inhibitory connections that a neuron receives from the upstream neurons and interneurons^51,65–67^. In particular, the diversity in STD of thalamocortical connections arriving at L4 neurons further contributes to the diversity of adaptation^64^.

## Materials and Methods

### Subjects and surgical procedures

A total of 36 male Wistar rats (4–6 weeks old) were used in this experiment. All methods were performed in accordance with the relevant guidelines and regulations and were approved by the Animal Experimentation Ethics Committee of the Australian National University (AEEC 2012/64; 2015/74). Animals were housed in a controlled environment with a 12-hour light–dark cycle. Anesthesia was induced by intraperitoneal administration of urethane (1.5 g/kg body weight). During the recording sessions, the level of anesthesia was regularly monitored by the hind paw and the corneal reflexes, and maintained at a stable level by administration of a top-up urethane injection (10% of the original dosage), if necessary. The rat head was fixed in a stereotaxic apparatus, a midline incision was made and the fascia was removed. A craniotomy was made above the left barrel cortex centered at 2.7 mm posterior and 5 mm lateral to the bregma. The dura mater was removed before inserting the recording pipette.

### Juxtacellular recording and labelling

Patch pipettes were pulled from borosilicate glass to reach impedance of 6–10 MΩ. The pipette was filled with rat ringer’s solution containing 2% neurobiotin. The pipette was positioned above the duratomy area, and lowered rapidly using a Sutter micromanipulator with high pressure (about 300 mm Hg) to pass the pial matter. The pressure was then dropped to 15–20 mmHg and the pipette was advanced at a speed of 2 µm/s while searching for neurons. Pipette resistance was constantly monitored using the current clamp mode of a BVC-700A amplifier (Dagan Corporation, Minneapolis, MN) and applying 1-nA current pulses with a duration of 200 ms at the frequency of 2.5 Hz. Upon observing fluctuations indicating close contact with a cell and a >4 fold increase in the pipette resistance, the pressure was removed and juxtacellular (loose cell-attached) recording was performed. At the end of the whisker stimulation protocol (see below), the neuron was loaded with neurobiotin by application of 1–5 nA current pulses of 200-ms duration at a frequency of 2.5 Hz^49^.

Across the experiments, we reconstructed 68 neurons. However, when more than one neuron was recorded/labelled in a single animal, the correspondence between the recorded neuron and the morphologically reconstructed ones was not always possible. Out of the total reconstructions, a subset of 45 neurons was identified for which a link between morphology and function could be made.

### Whisker stimulation protocol

A MATLAB (MathWorks, Inc., Natick, MA) script presented the stimuli and acquired the neuronal data through the analogue input and output of a data acquisition card (National Instruments, Austin, TX) at a sampling rate of 64 kHz. The whisker stimulation protocol was composed of discrete trials of 3.0-s duration, each of which containing a train of identical deflections at 2, 4, 8, 16 and 32 Hz. Each deflection was a brief (20 ms) biphasic vertical movement generated by a piezoelectric actuator. The principal whisker corresponding to the recorded neuron was identified manually and placed into a light cannula glued to the piezoelectric actuator. In addition to the regular trains of deflections, there was an irregular stimulus in which frequencies of 2, 4, 16, and 32 Hz were combined to produce a mean frequency of 8 Hz. The trials of regular stimulation along with the irregular stimulation were presented in a pseudorandom order with inter-trial intervals of 600-ms duration. Each stimulus was repeated for a minimum of 30 trials. A total of 147 neurons were recorded.

### Immunohistochemistry

To reconstruct the labeled neurons, the brain was removed and fixed in paraformaldehyde solution (4%). After gradual rehydration with PBS containing sucrose 10 to 30% W/V, the brain was sectioned with a Leica CM1580 cryostat at 120 µm thickness. The sections were penetrated using PBS solution containing %1 Triton X-100 (v/v, Sigma-Aldrich) for ~4 hours at room temperature and then washed with PBS. The slices were further incubated in PBS solution containing Alexa Fluor® 488 streptavidin conjugated secondary antibody (1:750 dilution, S11223, Thermo Fisher Scientific, Waltham, MA, USA) and maintained overnight in dark at 4 °C on a shaker. The brain sections were then washed and mounted on slides in a dark environment and the labeled neurons were visualized using an A1 Nikon confocal microscope and imaging system.

### Neuronal response analyses

The spikes corresponding to each trial were extracted by applying a threshold-crossing criterion onto the bandpass-filtered signal acquired continuously during the experiment. The extracted spikes were further visually inspected for waveform consistency.

Neuronal responses were characterized by counting the number of spikes generated in each trial over the window of interest. The net neuronal response to a stimulus was defined as the rate of spiking over the 3 s stimulus duration averaged across trials. Likewise, the cumulative neuronal response at a given time instance reports the average rate of spiking from the stimulus onset up to that time instance.

The responsiveness index was defined as the net neuronal response rate (measured over the whole stimulus train) divided by the rate of spiking in response to the first deflection. Likewise, the cumulative responsiveness index was defined as the ratio of cumulative neuronal response rate to the rate of spiking in response to the first deflection.

At each stimulation frequency, the latency of neuronal response to a given deflection was defined as the first 7-ms time bin for which the average response was higher than the baseline activity based on a random permutation test with a false positive rate of <0.05.

## Data Availability

Datasets supporting this article can be found at the central data repository of the Australian National University, see 10.4225/13/59a5f73786ca7.
