# Publisher Correction: Response dynamics of rat barrel cortex neurons to repeated sensory stimulation

**DOI:** 10.1038/s41598-018-33000-1

**Published:** 2019-03-06

**Authors:** Ehsan Kheradpezhouh, Mehdi Adibi, Ehsan Arabzadeh

**Affiliations:** 1 Eccles Institute of Neuroscience, John Curtin School of Medical Research, Australian National University, Canberra, ACT Australia; 2 Australian Research Council Centre of Excellence for Integrative Brain Function, Australian National University Node, Canberra, ACT Australia; 3 University of New South Wales, UNSW, Sydney, NSW Australia; 4 International School for Advanced Studies – SISSA, Trieste, Italy

Correction to: *Scientific Reports* 10.1038/s41598-017-11477-6, published online 13 September 2017

In the original PDF version of this Article an incorrect version of Figure 8 was published. This has now been corrected in the PDF; the HTML version of the paper was correct from the time of publication.

